# Characterization of SARS-CoV-2 Variants N501Y.V1 and N501Y.V2 Spike on Viral Infectivity

**DOI:** 10.3389/fcimb.2021.720357

**Published:** 2021-10-13

**Authors:** Haijun Tang, Long Gao, Zhao Wu, Fang Meng, Xin Zhao, Yun Shao, Xiaohua Shi, Shigang Qiao, Jianzhong An, Xiaohong Du, F. Xiao-Feng Qin

**Affiliations:** ^1^ Institute of Systems Medicine, Chinese Academy of Medical Sciences & Peking Union Medical College, Beijing, China; ^2^ Suzhou Institute of Systems Medicine, Suzhou, China; ^3^ Department of Gastroenterology, The Affiliated Suzhou Science and Technology Town Hospital of Nanjing Medical University, Suzhou, China; ^4^ Institute of Clinical Medicine Research, Suzhou Science and Technology Town Hospital, The Affiliated Suzhou Hospital of Nanjing Medical University, Suzhou, China

**Keywords:** SARS-CoV-2, N501Y.V1, N501Y.V2, infectivity, thermal stability

## Abstract

SARS-coronavirus 2 (SARS-CoV-2), pathogen of coronavirus disease 2019 (COVID-19), is constantly evolving to adapt to the host and evade antiviral immunity. The newly emerging variants N501Y.V1 (B.1.1.7) and N501Y.V2 (B.1.351), first reported in the United Kingdom and South Africa respectively, raised concerns due to the unusually rapid global spread. The mutations in spike (S) protein may contribute to the rapid spread of these variants. Here, with a vesicular stomatitis virus (VSV)-based pseudotype system, we demonstrated that the pseudovirus bearing N501Y.V2 S protein has higher infection efficiency than pseudovirus with wildtype (WT) and D614G S protein. Moreover, pseudovirus with N501Y.V1 or N501Y.V2 S protein has better thermal stability than WT and D614G, suggesting these mutations of variants may increase the stability of SARS-CoV-2 S protein and virion. However, the pseudovirus bearing N501Y.V1 or N501Y.V2 S protein has similar sensitivity to inhibitors of protease and endocytosis with WT and D614G. These findings could be of value in preventing the spread of virus and developing drugs for emerging SARS-CoV-2 variants.

## Introduction

The coronavirus disease 2019 (COVID-19) pandemic has become a great threat to the health of human beings. As of May 23, 2021, more than 160 million confirmed cases and approximately 3.4 million deaths have been reported worldwide (https://covid19.who.int). SARS-coronavirus 2 (SARS-CoV-2), pathogen of COVID-19, can cause infection of multiple organs throughout the body, mainly respiratory, digestive, circulatory, genitourinary and nervous systems. Although most symptoms of COVID-19 patients are mild, some may have severe pneumonia, even multiple organs failure, particularly in older people and those pre-existing chronic diseases (Lv et al., 2020).

SARS-CoV-2 uses spike (S) protein to bind host receptor angiotensin-converting enzyme 2 (ACE2), followed by membrane fusion and the release of viral RNA genome into the cytoplasm (Li et al., 2003; Benton et al., 2020; Shang et al., 2020; Wan et al., 2020; Zhou et al., 2020). As a type I membrane fusion protein, SARS-CoV-2 S protein composed of S1 and S2 subunits. S1 subunit mediates the attachment of virion to host cell, while S2 subunit mediates the fusion between viral and cellular membrane (Belouzard et al., 2009; Walls et al., 2020; Watanabe et al., 2020). Therefore, the S protein is essential to viral infectivity and transmissibility in the host (Hulswit et al., 2016). Before the membrane fusion, SARS-CoV-2 S protein needs to be cleaved by proteases, such as furin, cathepsins, trypsin, or transmembrane protease serine 2 (TMPRSS2). The cleavage of S protein by these proteases greatly affects the efficiency of virus infection on host cells (Palacios et al., 2020).

RNA viruses have a high mutation rate in the process of host transmission. Some mutations may alter viral pathogenicity, virulence, and/or transmissibility (Duffy, 2018). The mutations of SARS-CoV-2 S protein probably alter virus infectivity. In particular, D614G mutation was first identified in Europe, then replaced previously circulating lineages and led to a global pandemic (Korber et al., 2020). Studies show that the D614G substitution leads to the enhancement of viral replication and infectivity in human lung epithelial cells (Hou et al., 2020; Korber et al., 2020). Since December 2020, emergence of new SARS-CoV-2 variants N501Y.V1 (also known as B.1.1.7) in the United Kingdom and N501Y.V2 (also known as B.1.351) in South Africa (James et al., 2020; Kirby, 2021; Makoni, 2021; Rubin et al., 2021; Tegally et al., 2021) aroused widespread concern because of their extensive mutations and enhanced transmissibility.

The SARS-CoV-2 N501Y.V1 lineage has 17 non-synonymous mutations and deletions, and many of them locate in the S protein. N501Y.V2 lineage includes ten mutations in the S protein, and three of them are located in the receptor binding domain (RBD) (K417N, E484K and N501Y). As a common mutation of N501Y.V1 and N501Y.V2, N501Y mutation was first reported in a mouse model and hereafter identified in populations of many countries and regions (Gu et al., 2020; James et al., 2020; Kirby, 2021). In the mouse model, N501Y mutation enhances the binding affinity of SARS-CoV-2 S protein with mouse ACE2, and increase infectivity in mouse lung tissue (Gu et al., 2020). E484K mutation was first identified in N501Y.V2, and was reported to be associated with escaping from neutralizing antibodies (Baum et al., 2020; Weisblum et al., 2020). Interestingly, N501Y.V3 (also known as P.1) lineage in Brazil almost has the same three RBD mutations as N501Y.V2 lineage, except for the K417N/T substitution (Greaney et al., 2021). A701V, one of S protein mutation in N501Y.V2 lineage (Tegally et al., 2021), become the dominant strain in the third wave in Malaysia, and may affect the infectivity of the virus. In addition, the P681H substitution in SARS-CoV-2 S protein is adjacent to the furin cleavage site, implying the potential influence on the infection tropism and efficiency of virus (Millet and Whittaker, 2014; Hoffmann et al., 2020a).

SARS-CoV-2 N501Y.V1 and N501Y.V2 lineages rapidly replaced the original strain, suggesting the mutations of these variants may be related to increased transmissibility. Preliminary studies have shown that N501Y.V1 and N501Y.V2 lineages have high transmission efficiency and immune escape from neutralizing antibodies than the original strain (Galloway et al., 2021; Hoffmann et al., 2021; Roquebert et al., 2021). However, the transmission properties and biological characteristics of these variants remain elusive. In this study, we constructed pseudoviruses bearing SARS-CoV-2 S protein with mutations of N501Y.V1 and N501Y.V2 variants. We investigated the infectivity, S protein cleavage, thermal stability and drug inhibition properties of N501Y.V1 and N501Y.V2 pseudoviruses. This research promotes our understanding to the evolution of SARS-CoV-2 variants as well as the development of COVID-19 therapeutic drugs.

## Materials and Methods

### Construction of Expression Plasmids

Expression vector pcDNA3.1-2S (GenBank: MT_613044) with codon-optimized S gene of SARS-CoV-2 was obtained from Genscript Biotechnology Company. In order to effectively integrate S protein into pseudovirion, the last 19 amino acids of S protein were replaced with HA tag according to previously reported with modifications (Whitt, 2010; Zettl et al., 2020). The S genes with single-site mutation were obtained with the site-directed mutagenesis kit (KOD). To construct variants with multiple mutations, the plasmids were synthesized with ClonExpress Ultra One Step Cloning Kit (Vazyme). Briefly, primers containing site-directed mutation and homologous sequences were designed for PCR amplification to generate several fragments carrying different mutations, then these overlapping fragments were ligated together using homologous recombinase to re-assemble the expression vector with complete S gene coding sequence. All the resulting S protein expression vectors were verified by Sanger’s DNA sequencing at GenewiZ Biotechnology Co., Ltd. The sequencing results of SARS-CoV-2 S mutants are presented in **Supplementary Figures S1A–D**. The primers used for mutagenesis are listed in **Supplementary Tables S1**, **S2**.

### Cell Lines

Human cell lines (293T, Caco-2, Calu3, Huh7, H460, A549, NCI-H1299), Africa monkey kidney Vero cell, and mouse cell lines (MC38 and LLC-JSP) were obtained from American Type Culture Collection. Cells stably expressing human ACE2 and/or TMPRSS2 (293T-hACE2/293T-hACE2-TMPRSS2, Caco2-hACE2, A549-hACE2) were constructed with lentiviral mediated gene transduction. H460 cell line was cultured in RPMI (HyClone), and the others were cultured in DMEM (HyClone). All above cells were maintained in the medium containing 10% fetal bovine serum (FBS, Gibco) and 100 U/mL of Penicillin-Streptomycin solution (Gibco). The cells were cultured in an incubator with atmosphere of 5% CO_2_ at 37°C.

### Production and Quantification of SARS-CoV-2 Pseudovirions

Pseudovirions bearing SARS-CoV-2 S protein were generated according to published protocols (Nie et al., 2020b). Briefly, on the day before transfection, 5x10^6^ 293T cells were seeded into 10 cm cell culture dish. The next day, when the cells reached 70–90% confluent, 293T cells were transfected to express the S gene with lipofectamine 3000 (Invitrogen). 12 h post transfection, the cells were infected with G*△G-VSV dual reporter virus, a GP-deleted replication defective recombinant VSV virus containing firefly luciferase and eGFP reporter genes (kindly provided by UltraImmune Inc., Patent pending CN112760297A). 6 h post infection, the cell supernatant was discarded, and the cells were gently washed twice with PBS. Next, 10 mL fresh DMEM medium was added into the cell culture dish. 24 h post infection, the supernatant containing pseudovirions was harvested, then centrifuged at 3500 g for 5 min to remove the cell debris, and stored at -80°C in 1.5 mL aliquots until use.

Pseudovirions were quantified by RT-qPCR. Virus RNA was extracted from 200 μL of supernatant containing pseudovirions with Viral RNA/DNA Mini kit, and reversed to cDNA by PrimeScript™ RT reagent Kit (Takara). Pseudovirions quantification was performed using FastStart Essential DNA Green Master (Roche) following the manufacturer’s protocols. The copy numbers of pseudovirions were calculated by the VSV-P protein gene. Primers are listed in **Supplementary Table S3**.

### Infection Assay

Before the infection assay, the pseudovirions were normalized to the same amount by quantitative RT-qPCR. The day before virus infection, 2x10^4^/100 μL cells were seeded into 96-well plates. The next day, cells were infected with 100 μL of media containing pseudovirions. 16 or 24 h post infection, cells were lysed with 60 μL diluted passive lysis buffer (Promega) at room temperature for 10 min. The infection efficiency of the pseudovirus was detected by measuring the activity of luciferase using Luciferase Assay System (Promega). For analysis of pseudovirus entry into 293T-hACE2 cells at indicated time points, images were captured in each well with a Nikon inverted microscope ECLIPSE Ts2 epifluorescence microscope. Each group contained 4 replicates, and each experiment was repeated at least two times.

### Thermal Stability Assay of SARS-CoV-2 Pseudovirions

Pseudovirions incorporated with S protein of SARS, SARS-CoV-2 WT, D614G, N501Y.V1 and N501Y.V2 lineages were diluted with DMEM and incubated in a 37°C or 42°C water bath. At indicated time points, 100 μL DMEM containing the pseudovirions was taken out and added into the wells with 293T-hACE2. 24 h post infection, the infection efficiency of pseudovirus was detected by measuring the activity of luciferase. The relative infection efficiency of pseudovirus was calculated by dividing the relative luminescence unit (RLU) values at each time point by the mean viral RLU at 0 h.

### SARS-CoV-2 S Expression and Incorporation Assay

In order to verify the expression of S protein in cells, the SARS-CoV-2 S expression vectors were transfected into 293T cells with lipofectamine 3000 (Invitrogen). 40 h post transfection, cells were lysed by RIPA Lysis Buffer (Beyotime) for 30 min on ice. In order to analyze incorporation of S protein into pseudotyped particles, VSV pseudovirions was concentrated with PEG8000. Briefly, medium containing pseudovirions was loaded by 6% PEG8000 and 0.1M NaCl, and shaked on the ice overnight. Next, the mixture was centrifugated (10,000 g) for 120 min at 4°C. After that, the supernatant was discarded, and the residual volume was mixed with 50 uL RIPA.

All the samples were heated at 96°C for 10 minutes, and then the expression of S protein was detected by SDS-PAGE. After protein transfer, the nitrocellulose membrane was blocked by 5% milk for 1 h. Then the membranes were blotted with primary antibodies, followed by incubation with a secondary antibody and visualized by Bio-Rad ChgmiDoc MP Bio-Rad Laboratorigs. The primary antibodies used for western blot analysis were as follows: mouse anti-SARS-CoV/SARS-CoV-2 (COVID-19) spike [1A9] (Genetex, 1:2000), mouse anti-β-actin monoclonal antibody (TransGen Biotech, 1:2000), mouse anti-VSV matrix protein (Kerafast, 1:2500). We used a horseradish peroxidase-linked anti-mouse IgG antibody (Cell Signaling Technology, 1:5000) as secondary antibody.

### Effects of Endocytosis and Protease Inhibitors on Pseudovirion Entry

For experiments involving endocytosis and protease inhibitors, 293T-hACE2 or 293T-hACE2-TMPRSS2 cells were pretreated with either endocytosis inhibitors (Chloroquine, MedChemExpress, 0-10 μM; Tetradrine, Sigma-Aldrich, 0-2 μM and Apilimod, MedChemExpress, 0-100 nM) or protease inhibitors (E64d, MedChemExpress, 0-5 μM; Camostat, MedChemExpress, 0-100 μM) for 2 h. Then the pseudovirions was added into the corresponding wells of plates. 24 h post infection, cells were lysed, and the infection efficiency of viruses was detected by measuring the luciferase activity using Luciferase Assay System.

### Statistical Analysis

The GraphPad Prism 7 software package was used for all statistical analyses. The unpaired two-tails Student’s t-test was used to determine the statistical significance. The values were presented as mean ± SEM and p < 0.05 was defined as statistically significant [ns represents no significant difference, p < 0.05 (*), p < 0.01 (**), p < 0.001 (***), p < 0.0001 (****)].

## Results

### The Expression and Pseudovirion Incorporation of SARS-CoV-2 S Protein

**Figure 1:**
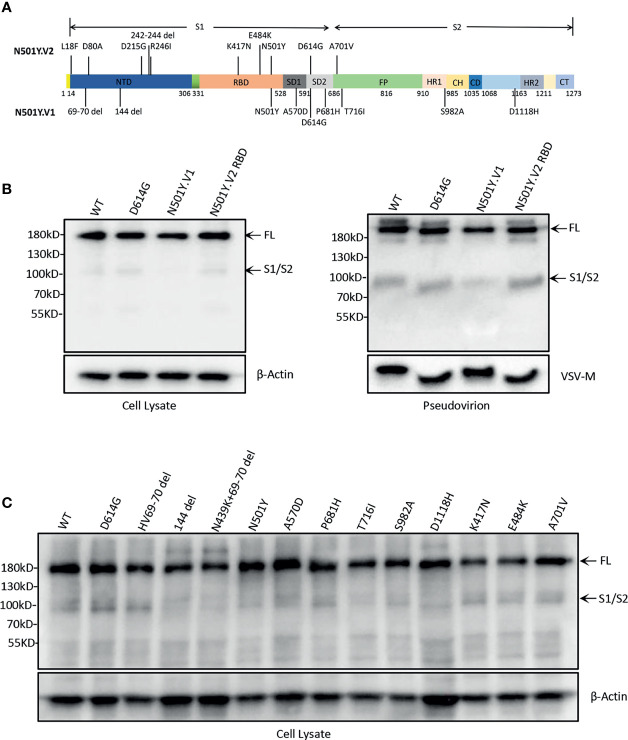
SARS-CoV-2 variants spike protein expression and incorporation into pseudovirions. **(A)** Construction of SARS-CoV-2 variants N501Y.V1, N501Y.V2, and N501Y.V2 RBD S protein. The S protein of N501Y.V1 include nine mutations (HV69-70 del, 144 del, N501Y, D614G, P681H, T716I, S982A, and D1118H), N501Y.V2 include ten mutations (L18F, D80A, D215G, 242-244 del, R246I, K417N, E484K, N501Y, D614G and A701V) and N501Y-V2 RBD include D614G and three major mutations in the RBD (K417N, E484K and N501Y). **(B)** Analysis of S protein expression and particle incorporation of N501Y.V1 and N501Y.V2 RBD lineages. The S proteins expression and incorporation to pseudovirion were measured by western blot using antibody against spike. β-Actin (cell lysates) and VSV-M (particles) served as loading controls. Full-length S protein and S1/S2 cleavage are annotated. Shown are representative blots from three experiments. **(C)** Analysis of the S protein expression of single-site mutation of spike protein in N501Y.V1 and N501Y.V2 lineages by western blot. The S proteins expression were detected in cell lysate by western blot using antibody against S protein, and β-Actin served as loading controls.

The purpose of this study was to gain insights into the differences of N501Y.V1 and N501Y.V2 lineages with previous WT and D614G. For this, we first constructed S genes expression vectors of N501Y.V1 variant (with all nine mutations) and N501Y.V2 RBD mutations (K417N, E484K, N501Y and D614G) (**Figure 1A**). Then, the S protein expression in 293T cells was detected by western blot. There were two major bands, 180 kDa band (full-length S protein) and 90 kDa band (cleaved S protein, S2 subunit). The full-length S protein was observed in cells, but cleaved S protein was not obvious (**Figure 1B**). We also detected the S protein expression of single-site mutation in N501Y.V1 and N501Y.V2 RBD. The differences in cleavage of S protein with point mutations were also not observed (**Figure 1C**).

The efficiency of S protein incorporation into pseudovirions was analysed by western blot using antibody against SARS-CoV-2 spike and VSV-M. The results indicated that all four S proteins successfully incorporate into pseudovirions, and the cleaved S protein (S2 subunit) is at the similar level, except for a slightly weakened band in N501Y.V1 (**Figure 1B**).

### Infectivity of SARS-CoV-2 Variants N501Y.V1 and N501Y.V2 Lineages

**Figure 2:**
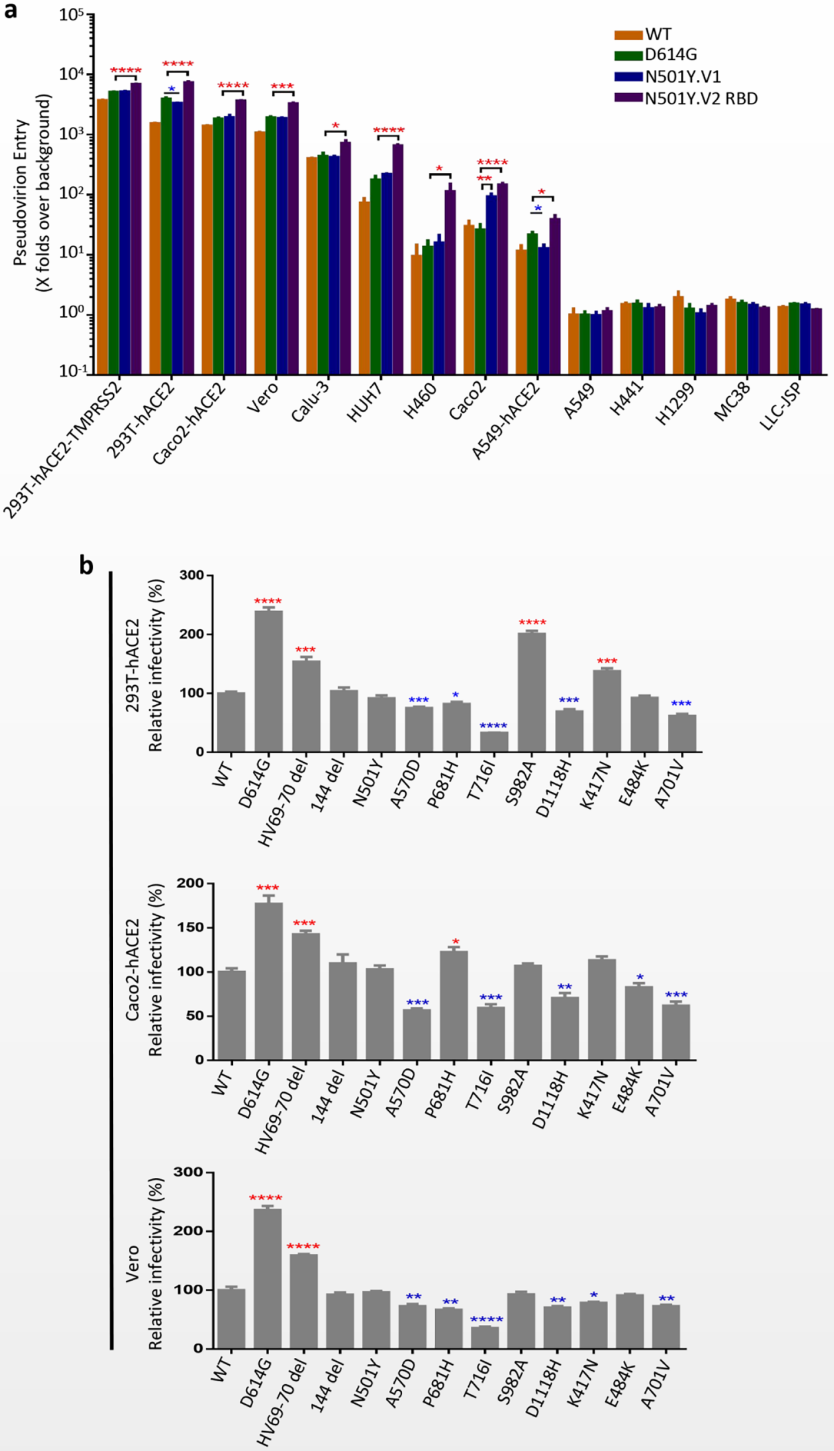
Infection efficiency of SARS-CoV-2 variants N501Y.V1 and N501Y.V2 in mammalian cell lines. **(A)** Infection efficiency of SARS-CoV-2 WT, D614G, N501Y.V1 and N501Y.V2 RBD variants in human and animal cell lines. **(B)** Infectivity analysis of N501Y.V1 and N501Y.V2 RBD single-site mutants. N501Y.V1 and N501Y.V2 RBD single-site mutants conducted in 293T-hACE2 (top), Caco2-hACE2 (middle) and Vero (bottom). At 24 h postinoculation, transduction efficiency was analyzed by luciferase activity in cell lysates. The infection efficiency of viruses was calculated by dividing the RLU values by the mean SARS-CoV-2 WT RLU. Experiments were done in 4 replicates and repeated at least twice. One representative is shown with error bars indicating SEM. The significant changes were marked with colored symbols, red stars for increased, blue stars for decreased. ns represents no significant difference, p < 0.05 (*), p < 0.01 (**), p < 0.001 (***), p < 0.0001 (****).

**Figure 3:**
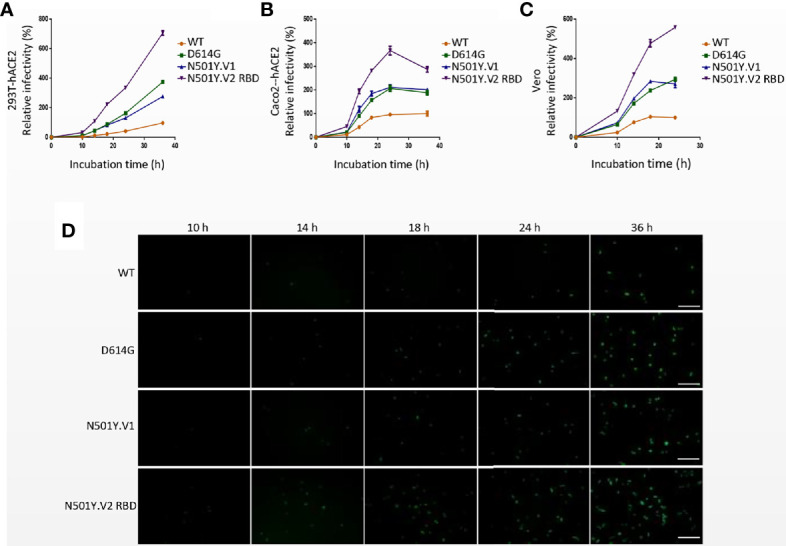
Infection efficiency of SARS-CoV-2 variants N501Y.V1 and N501Y.V2 RBD in target cells at different time points. **(A–C)** Infectivity analysis of SARS-CoV-2 WT, D614G, N501Y.V1 and N501Y.V2 RBD entry into 293T-hACE2 **(A)**, Caco2-hACE2 **(B)**, and Vero **(C)** cells at 10 h, 14 h, 18 h, 24 h, and 36 h. Transduction efficiency was analyzed by luciferase activity in cell lysates. The infection efficiency of viruses was calculated by dividing the RLU values at each time point by the average SARS-CoV-2 WT RLU values at 36h. Experiments were done in 4 replicates and repeated at least twice. One representative is shown with error bars indicating SEM. **(D)** Representative images of SARS-Cov-2 WT, D614G, N501Y.V1 and N501Y.V2 RBD entry into 293T-hACE2 cells at indicated time points. The scale bar indicates 200 µm.

Replication-defective VSV pseudovirus incorporated with S protein from coronavirus faithfully reflect the key aspects of virus entry into host cells, and have been applied in neutralizing antibody quantification and entry mechanism study of SARS-CoV-2 (Kleine-Weber et al., 2019; Condor Capcha et al., 2020; Nie et al., 2020a; Nie et al., 2020b). Moreover, several monoclonal antibodies targeting S protein of SARS-CoV-2 could completely inhibit the infection of VSV-based pseudovirus (data not shown). To determine the infectivity of N501Y.V1 and N501Y.V2 variants, a panel cell lines originated from human and animals were infected with VSV pseudoviruses of SARS-CoV-2 WT, D614G, N501Y.V1 and N501Y.V2 RBD (**Figure 2A**). All four pseudoviruses could efficiently infect certain cell lines, including 293T-hACE2-TMPRSS2, 293T-hACE2, Huh-7, Caco-2, Caco2-hACE2, Vero, Calu-3, A549-hACE2 and H460. Compared with WT, pseudovirirus bearing S protein with D614G mutation (including D614G, N501Y.V1 and N501Y.V2 RBD) were significantly more infectious in several susceptible cells. More importantly, N501Y.V2 RBD pseudovirus has higher infection efficiency than D614G in all susceptible cells, while N501Y.V1 has no obviously difference. Next, we detected the infectivity of N501Y.V2 pseudovirus to different cells (**Supplementary Figures S2A–E**). Compared with D614G, N501Y.V2 pseudovirus had significant difference in the infection efficiency of 293T-hACE2-TMPRSS2, Caco2 and Caco2-hACE2 cells. However, the infection efficiency of N501Y.V2 RBD pesudovirus is higher than N501Y.V2 in some susceptible cells. In addition, we detected the infection efficiency of SARS-CoV-2 WT, D614G, N501Y.V1 and N501Y.V2 RBD in target cells at 10 h, 14 h, 18 h, 24 h, and 36 h. The infection rate of N501Y.V2 at different time point was also higher than that of D614G (**Figures 3A–D**).

We also tested the infectivity of pseudoviruses with single-site S protein mutations in 293T-hACE2, Caco2-hACE2 and Vero cells (**Figure 2B**). Compared with WT pseudovirus, the viral infectivities of D614G and HV69-70 deletion were increased in all three cells. In contrast, T716I, A570D, D118H and A701V mutations caused a modest reduction in viral infectivity. It is worth noting that the N501Y mutation does not result in a significant change in the infectivity of the pseudovirus.

### Thermal Stability Analysis of N501Y.V1 and N501Y.V2 Pseudovirions

**Figure 4:**
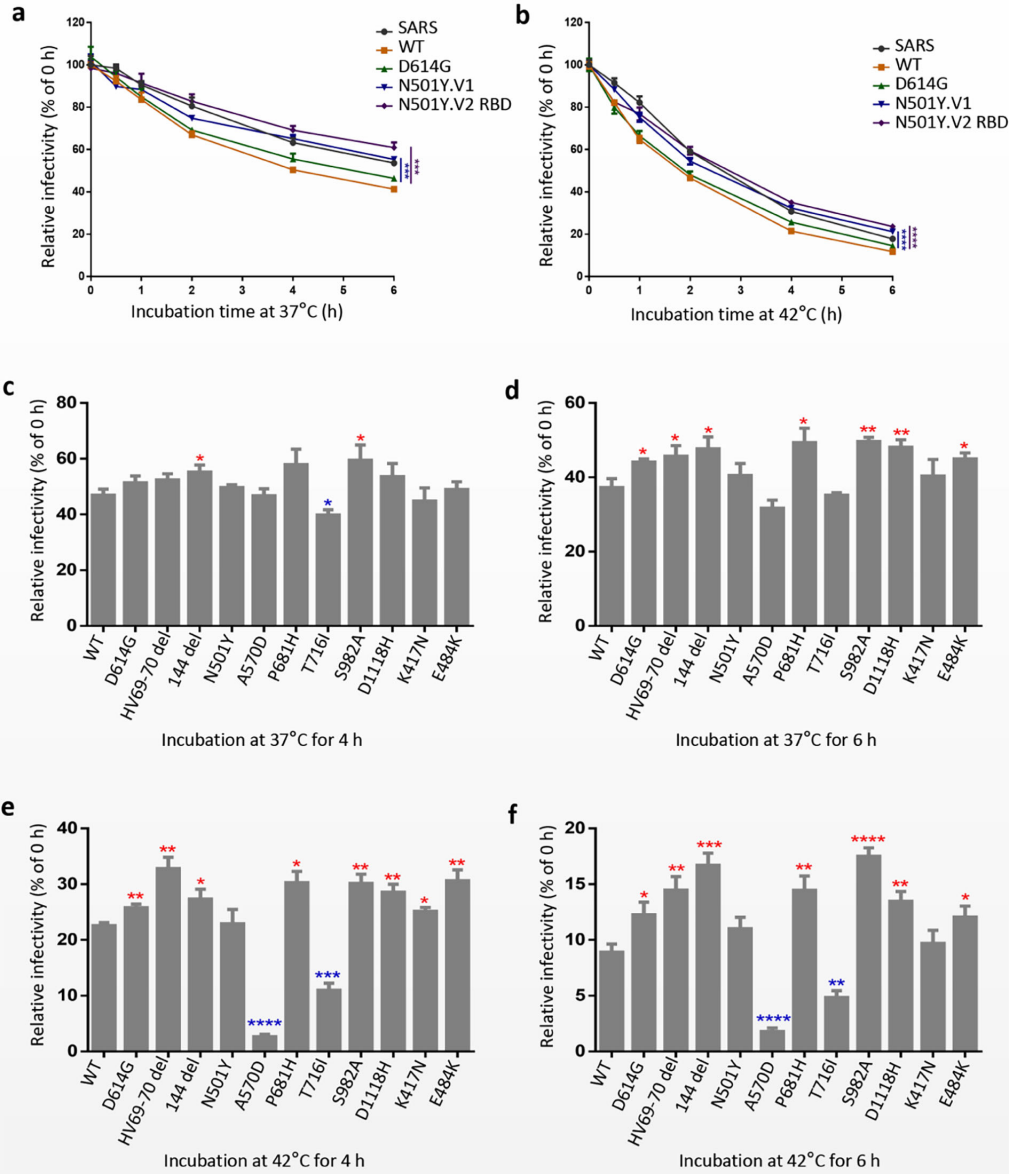
SARS-CoV-2 variants N501Y.V1 and N501Y.V2 RBD are more thermal stable than WT and D614G. **(A, B)** SARS-CoV, SARS-CoV-2 WT, D614G, N501Y.V1 and N501Y.V2 RBD S pseudovirions were incubated in cell culture medium DMEM at 37°C **(A)** or 42°C **(B)** for the specified times (0 to 6 h). The viruses were quantified for their infectious levels by luciferase on 293T-hACE2 cells. The infection efficiency of remaining viruses was normalized by the average fluorescence values at 0h. **(C–F)** The single-site mutations of N501Y.V1 and N501Y.V2 RBD S pseudovirions were incubated in cell culture medium DMEM at 37°C for 4 h **(C)** and 6 h **(D)** or 42°C for 4 h **(E)** and 6 h **(F)**. The experiments were repeated twice, and means with SEM are shown. The significant changes were marked with colored symbols, red stars for increased, blue stars for decreased. ns represents no significant difference, p < 0.05 (*), p < 0.01 (**), p < 0.001 (***), p < 0.0001 (****).

In order to study the effect of S mutations of N501Y.V1 and N501Y.V2 lineages on the stability of the virus, we measured the infectivity decay of pseudoviruses incorporated S protein of SARS, SARS-CoV-2 WT, D614G N501Y.V1 and N501Y.V2 RBD overtime at 37°C (normal body temperature) and 42°C (possible body temperature in fever). The infectivity of the five pseudoviruses decreased faster at 42°C than 37°C (**Figures 4A, B**). It is worth noting that pseudovirions bearing S protein of N501Y.V1 and N501Y.V2 RBD variants retained higher infectivity than WT and D614G at any temperature (**Figures 4A, B**). In addition, we detected the thermal stability of N501Y.V2 pseudovirus at 37°C and 42°C (**Supplementary Figures S3A–D**). Similar to N501Y.V2 RBD mutation, the stability of N501Y.V2 pseudovirus was significantly higher than that of WT and D614G.

We also examined the effect of single-site mutations of N501Y.V1 and N501Y.V2 lineages on the thermal stability of SARS-CoV-2. We measured the infectivity decay of pseudovirions bearing single-site mutations at 37°C or 42°C (**Figures 4C–F**). The results indicated that pseudovirions bearing HV69-70 deletion, 144 deletion, E484K, D614G, P681H, S982A or D1118H single-site mutations were more stable than SARS-CoV-2 WT, whereas A570D and T716I mutations decrease the stability of SARS-CoV-2 pseudovirion. Our findings show that, the S protein mutations of N501Y.V1 and N501Y.V2 variants may increase the conformational stability of spike to promote the infectivity of SARS-CoV-2 virion.

### Effect of Cathepsin Inhibitors and Serine Protease Inhibitor on Virus Entry

**Figure 5:**
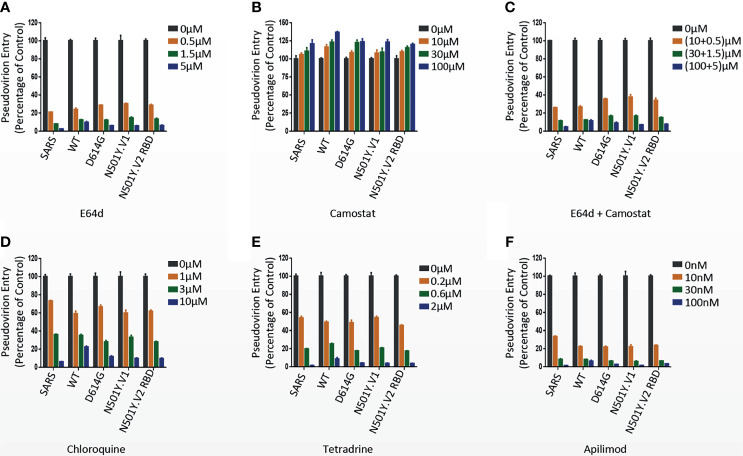
Effects of endocytosis and protease inhibitors on pseudovirion entry. **(A–C)** N501Y.V1 and N501Y.V2 RBD entry into host cells through CatB/L or TMPRSS2 activation was evaluated by adding E64d, Camostat or the combination of them (E64d + Camostat) to 293T-hACE2 cells 2 h prior to transduction. The luciferase activity was measured 24 h post transduction. **(D–F)** Inhibition of entry of SARS-CoV, SARS-CoV-2 WT, D614G, N501Y.V1 and N501Y.V2 RBD by adding endocytosis inhibitors Chloroquine, Tetradeine, and Apilimod to 293T-hACE2 cells 2 h prior to transduction. Experiments were done in 4 replicates and repeated at least twice. One representative is shown with error bars indicating SEM.

The endosomal cysteine proteases cathepsin B and L (CatB/L) and the serine protease TMPRSS2 were required for coronavirus S protein priming in target cells (Simmons et al., 2005; Glowacka et al., 2011). We next investigated the effect of S protein mutations in N501Y.V1 and N501Y.V2 on protease dependent entry in 293T-hACE2 (TMPRSS2^-^) and 293T-hACE2-TMPRSS2. Cathepsins inhibitor E64d treatment absolutely inhibited the infection of WT, D614G, N501Y.V1 and N501Y.V2 RBD pseudoviruses into 293T-hACE2 cells (**Figure 5A** and **Supplementary Figures S4A, B**), indicating that CatB/L is the dominant proteases required for priming of SARS-CoV-2 S protein in TMPRSS2^-^ cells. Compared with 293T-hACE2 cells, the inhibition efficiency of E64d was attenuated in 293T-hACE2-TMPRSS2 cells (**Supplementary Figures S4A, B**). Camostat, a serine protease inhibitor, cannot inhibit SARS-CoV-2 entry into 293T-hACE2 cells (**Figures 5B, C**), while partially reduced SARS-CoV-2 entry into 293T-hACE2-TMPRSS2 cells (**Supplementary Figure S4B**). Moreover, the combined inhibitory effect of E64d and Camostat in 293T-hACE2-TMPRSS2 cells was more obvious than any of them alone (**Supplementary Figure S4B**). These results indicated that Cat B/L is required for priming of S protein both in TMPRSS2^-^ and TMPRSS2^+^ 293T cells, while TMPRSS2 is required only in TMPRSS2^+^ 293T cells. More importantly, Camostat had a more significant inhibitory effect on N501Y.V1 and N501Y.V2-RBD pseudoviruses than D614G (**Supplementary Figure S4B**), indicating that the priming of N501Y.V1 and N501Y.V2-RBD S protein maybe more dependent on TMPRSS2 activities.

### SARS-CoV-2 Enters Cell Lines Through Endocytosis

Previous studies have shown that SARS-CoV-2 enters target cells mainly through endocytosis (Palacios et al., 2020). Next, we evaluated whether N501Y.V1 and N501Y.V2 lineages enter into host cells through endocytosis. 293T-hACE2 and 293T-hACE2-TMPRSS2 cells were pretreated with endocytosis inhibitors Chloroquine, Tetradeine, and Apilimod, and then their effect on virus entry was evaluated. Consistent with the previous study, all three inhibitors decreased the infection of WT, D614G N501Y.V1 and N501Y.V2 RBD pseudovirions in a dose-dependent manner (**Figures 5D–F** and **Supplementary Figures S4C, D**). Compared with 293T-hACE2 cells, the inhibition efficiency of the three inhibitors in 293T-hACE2-TMPRSS2 cells was also attenuated (**Supplementary Figures S4C, D**). However, compared with WT and D614G, the inhibition efficiency of these inhibitors on N501Y.V1 and N501Y.V2 RBD lineages has no significant difference (**Supplementary Figures S4C, D**). These results indicated that entry of N501Y.V1 and N501Y.V2-RBD pseudovirions into host cells mainly depend on the endocytosis in 293T-hACE2 cells, but presence of TMPRSS2 attenuate this dependence.

## Discussion and Conclusion

As RNA virus, SARS-CoV-2 has a high mutation rate during transmission in the population. Moreover, some mutations are adaptive because they alter pathogenesis, virulence, and/or transmissibility (Zhou et al., 2020). Particularly, the D614G mutation in the spike leads to increased virus infectivity and subsequently spread rapidly in the world, thereby gradually replacing previously circulating lineages (Hou et al., 2020; Korber et al., 2020; Li et al., 2020; Plante et al., 2020). In December 2020, newly emerging SARS-CoV-2 N501Y.V1 and N501Y.V2 variants are raising concerns in the UK and South Africa respectively, due to the enhancive spread ability of the virus. It is believed that mutations of N501Y.V1 and N501Y.V2 variants possibly change their immunogenicity and virus infectivity (Wang et al., 2021; Wibmer et al., 2021). In the present study, using a VSV-based pseudotyped system, we packaged pseudovirions bearing S protein with combination and single-site mutations of N501Y.V1 and N501Y.V2 variants. In this study, we investigated the infectivity, S protein cleavage, thermal stability and drug inhibition properties of the N501Y.V1 and N501Y.V2 lineages.

Our results demonstrated that the mutations of N501Y.V2 lineage enhanced the infection efficiency of SARS-CoV-2 in several susceptible cell lines. Interestingly, the infection efficiency of N501Y.V2 RBD pesudovirus is higher than N501Y.V2, implying the non-RBD S mutations of N501Y.V2 have negative effect on the infection of SARS-CoV-2. Recently, Li et al. (2021) showed that the infectivity of pseudovirions incorporated with N501Y.V2 S proteins was not obviously changed in human cells, while N501Y.V2 RBD mutations slightly but obviously increase the viral infection efficiency, which is consistent with our research. Previous studies in mice have shown that N501Y can increase the affinity for the mouse ACE2 receptor (Gu et al., 2020), which proves that this new variant can enhance its infectivity in the host. Kuzmina et al. (Kuzmina et al., 2021) reported that pseudovirions with N501Y+K417N or N501Y+E484K exhibited higher infection rates than the N501Y pseudovirion alone. Indeed, N501Y.V3 lineage includes three mutations in the RBD region (K417T, E484K and N501Y), and it almost has the same three mutations present in RBD as N501Y.V2 lineage, except for K417N/T substitution. These three RBD mutations of S protein maybe cause the higher infection efficiency of N501Y.V2 and N501Y.V3 *via* promoting the binding affinity with ACE2 (Khan et al., 2021). Therefore, the mutation sites of N501, E484 and K417 in RBD region are essential for virus infectivity. Compared with D614G, N501Y.V1 lineage had no significant change in infectivity. In 293T-hACE2 cells, the infection efficiency of N501Y.V1 was slightly lower than D614G; however, in cells expressed TMPRSS2, such as 293T-hACE2-TMPRSS2 and Caco-2 cells, its infectivity increased. These results indicate that the infection of N501Y.V1 lineage may be more dependent on TMPRSS2 activity. The effects of single-site mutations of N501Y.V1 and N501Y.V2 lineages on viral infectivity were variable. Pseudovirion with HV69-70 deletion could enhance the infectivity of the virus, while single-site mutation T716I, A570D, D118H, and A701V caused a modest reduction in viral infectivity. So, the infectivity of SARS-CoV-2 may be associated with the synergistic effects of different mutations, particularly the mutations in RBD region of S protein.

Priming of SARS-CoV S proteins by target cell proteases is crucial for virus entry into cells (Glowacka et al., 2011; Li, 2015). SARS-CoV-2 spike protein could be cleaved in cells, and the cleaved S protein was incorporated into VSV pseudovirions (Hoffmann et al., 2020b). In our study, the S protein of SARS-CoV-2 and its mutations were weakly cleaved in cells, and there was no significant difference between these mutants. Previous studies show that the cleavage efficiency of S protein into S1/S2 regulates virus infection (Hoffmann et al., 2020a). We compared the cleavage of S protein in WT, D614G, N501Y.V1 and N501Y.V2 RBD pseudovirions. To our surprise, the cleaved S protein band was slightly weakened in particles of N501Y.V1 than WT and D614G (**Figure 1B**). However, N501Y.V2 lineage, which is more infectious in cell lines, had no substantial differences in spike cleavage than WT and D614G virions, suggesting that the enhanced virus infectivity of N501Y.V2 lineage is not likely due to the difference of S protein cleavage.

It is reported that SARS-CoV-2 D614G mutant has a higher thermal stability than WT virions, and its strong infectivity and transmissibility may be related to the stability of the virus (Plante et al., 2020). However, it is reported that there is no significant difference among the stability of N501Y.V1, N501Y.V2 and N501Y.V3 pseudovirus, which is incubated at 33℃ for a period of time (Hoffmann et al., 2021). To better detect the thermal stability of SARS-CoV-2 variants virions in the host, we choose 37°C (normal body temperature) and 42°C (possible body temperature in fever) for treating virus. Importantly, we found that N501Y.V1 and N501Y.V2 lineages are more stable than D614G, indicating that the enhanced infectivity of N501Y.V1 and N501Y.V2 lineages in the population may be partly correlated with its higher thermal stability in the host and environment. The inconsistency between the research results is probably caused by the incubation temperature. The pseudovirion will be more unstable at 37°C and 42°C than 33°C, making the difference in thermal stability manifest. Moreover, the thermal stability of N501Y.V2 pesudovirus is higher than N501Y.V2 RBD, implying the non-RBD S mutations of N501Y.V2 have positive effect on the thermal stability of SARS-CoV-2. We further evaluated the effect of single-site mutations of N501Y.V1 and N501Y.V2 lineages on the stability of the virus. We found that several single-site mutations of N501Y.V1 and N501Y.V2 lineages can increase/decrease the thermal stability of pseudovirion. Therefore, the higher stability of N501Y.V1 and N501Y.V2 lineages may be related to the synergistic effects of different mutations.

SARS-CoV S protein is primed by the endosomal cysteine CatB/L protease in TMPRSS2^-^ cells (Simmons et al., 2005; Zhou et al., 2016). However, S protein priming by TMPRSS2 is also critical for viral entry into host cells (Kawase et al., 2012; Zhou et al., 2015; Iwata-Yoshikawa et al., 2019). Recent studies show that SARS-CoV-2 entry target cells depends on CatB/L and/or TMPRSS2 activity (Iwata-Yoshikawa et al., 2019; Hoffmann et al., 2020b; Hoffmann et al., 2021). The present study indicates that CatB/L is required for all SARS-CoV-2 variants entry in TMPRSS2^-^ cells, while TMPRSS2 is required for priming of all SARS-CoV-2 variants entry into TMPRSS2^+^ cells. More importantly, Camostat has a better inhibition effect on N501Y.V1 and N501Y.V2 lineages than D614G in TMPRSS2^+^ cells, indicating that the priming of N501Y.V1 and N501Y.V2 S protein maybe more dependent on TMPRSS2 activity.

In this study, we demonstrated that the infectivity of SARS-CoV-2 N501Y.V2 was increased in susceptible cells, and the enhanced virus infectivity was not likely related to the cleavage of S protein. We further found that SARS-CoV-2 N501Y.V1 and N501Y.V2 mutants are more thermal stable in the host and environment, which may be associated with the rapid transmission of the two variants in the population. Finally, we proved that SARS-CoV-2 WT, D614G, N501Y.V1 and N501Y.V2 entry into host cells mainly through endocytosis, while both cathepsin and TMPRSS2 play important roles in virus entry. However, in view of the limitations of pseudoviruses and immortalized cell lines used in this study, our findings still need to be verified using infectious live virus and primary cells or tissues in future studies. In conclusion, our study plays a vital role in understanding the evolution and infectivity of SARS-CoV-2 variants, as well as the development of COVID-19 therapeutic drugs.

## Data Availability Statement

The original contributions presented in the study are included in the article/**Supplementary Material**. Further inquiries can be directed to the corresponding authors.

## Author Contributions

HT performed the experiments and wrote the manuscript. HT and XD analyzed data. LG, ZW, FM, XZ, YS, XS, SQ, and JA contributed to revise the manuscript and approved the final manuscript. XD and FQ was responsible for research design, strategy and supervision. All authors contributed to the article and approved the submitted version.

## Funding

This work was supported by: The National Natural Science Foundation of China (Grant 81701567, 81773058 and 31800726); The Chinese Academy of Medical Sciences Initiative for Innovative Medicine (Grant CAMS-I2M, 2016-I2M-1-005); National grand Foreign Experts projects (G20190001633 and G20190001639).

## Conflict of Interest

The authors declare that the research was conducted in the absence of any commercial or financial relationships that could be construed as a potential conflict of interest.

## Publisher’s Note

All claims expressed in this article are solely those of the authors and do not necessarily represent those of their affiliated organizations, or those of the publisher, the editors and the reviewers. Any product that may be evaluated in this article, or claim that may be made by its manufacturer, is not guaranteed or endorsed by the publisher.

## Supplementary Material

Supplementary Figure 1 DNA sequencing results of S gene mutations of N501Y.V1, N501Y.V2-RBD and N501Y.V2. SARS-CoV-2 S genes were sequenced with Sanger’s DNA sequencing at GenewiZ Biotechnology Co., Ltd. The DNA sequences of SARS-CoV-2 S mutants were compared with SARS-CoV-2 WT S gene with SnapGene software, and the related nucleotide mutation sites were marked in red boxes.

Supplementary Figure 2 Infection efficiency of SARS-CoV-2 N501Y.V2 in mammalian cell lines. Infection efficiency of SARS-CoV-2 WT, D614G, N501Y.V2 and N501Y.V2 RBD variants in 293T-hACE2 **(A)**, Vero **(B)**, 293T-hACE2-TMPRSS2 **(C)**, Caco2 **(D)**, Caco2-hACE2 **(E)** cells. ns represents no significant difference, p < 0.05 (*), p < 0.01 (**), p < 0.001 (***), p < 0.0001 (****).

Supplementary Figure 3 SARS-CoV-2 N501Y.V2 is more thermal stable than WT and D614G. SARS-CoV-2 WT, D614G, N501Y.V2 and N501Y.V2 RBD S pseudovirions were incubated in cell culture medium DMEM at 37°C for 4 h **(A)** and 6 h **(B)** or 42°C for 4 h **(C)** and 6 h **(D)**. The viruses were quantified for their infectious levels by luciferase on 293T-hACE2 cells. The infection efficiency of remaining viruses were normalized by the average fluorescence values at 0 h. ns represents no significant difference, p < 0.05 (*), p < 0.01 (**), p < 0.001 (***), p < 0.0001 (****).

Supplementary Figure 4 The effects of protease and endocytosis pathway inhibitors on the entry of SARS-CoV-2 N501Y.V1 and N501Y.V2 RBD. **(A, B)** Effect of CatB/L or TMPRSS2 on SARS-CoV-2 N501Y.V1 and N501Y.V2-RBD entry into host cells. E64d (0.4 μM), Camostat (50 μM) or the combination of them (E64d (0.4 μM) + Camostat (50 μM)) were added into 293T-hACE2 **(A)** or 293T-hACE2-TMPRSS2 **(B)** cells 2 h prior to transduction. The luciferase activity was measured 24 h post transduction. **(C, D)** Effect of endocytosis on SARS-CoV-2 N501Y.V1 and N501Y.V2 RBD entry into host cells. Endocytosis inhibitors Chloroquine (1 μM), Tetradeine (0.2 μM), and Apilimod (5 nM) were added into 293T-hACE2 **(C)** or 293T-hACE2-TMPRSS2 **(D)** cells 2 h prior to transduction. Experiments were done in 4 replicates and repeated at least twice. One representative is shown with error bars indicating SEM. ns represents no significant difference, p < 0.05 (*), p < 0.01 (**), p < 0.001 (***), p < 0.0001 (****).
