# Corrigendum: Characterization of SARS-CoV-2 Variants N501Y.V1 and N501Y.V2 Spike on Viral Infectivity

**DOI:** 10.3389/fcimb.2021.813645

**Published:** 2021-11-29

**Authors:** Haijun Tang, Long Gao, Zhao Wu, Fang Meng, Xin Zhao, Yun Shao, Xiaohua Shi, Shigang Qiao, Jianzhong An, Xiaohong Du, F. Xiao-Feng Qin

**Affiliations:** ^1^ Institute of Systems Medicine, Chinese Academy of Medical Sciences & Peking Union Medical College, Beijing, China; ^2^ Suzhou Institute of Systems Medicine, Suzhou, China; ^3^ Department of Gastroenterology, The Affiliated Suzhou Science and Technology Town Hospital of Nanjing Medical University, Suzhou, China; ^4^ Institute of Clinical Medicine Research, Suzhou Science and Technology Town Hospital, The Affiliated Suzhou Hospital of Nanjing Medical University, Suzhou, China

**Keywords:** SARS-CoV-2, N501Y.V1, N501Y.V2, infectivity, thermal stability

## Incorrect Affiliation

In the published article, there was an error in affiliation 1. Instead of “^1^Center of Systems Medicine, Institute of Basic Medical Sciences, Chinese Academy of Medical Sciences and Peking Union Medical College, Beijing, China” it should be “^1^Institute of Systems Medicine, Chinese Academy of Medical Sciences & Peking Union Medical College, Beijing, China”.

We apologize for these errors and state that these do not change the scientific conclusions of the article in any way. The original article has been updated.

## Publisher’s Note

All claims expressed in this article are solely those of the authors and do not necessarily represent those of their affiliated organizations, or those of the publisher, the editors and the reviewers. Any product that may be evaluated in this article, or claim that may be made by its manufacturer, is not guaranteed or endorsed by the publisher.

