# Supplementary figures and images for: Characterization of SARS-CoV-2 Variants N501Y.V1 and N501Y.V2 Spike on Viral Infectivity
Source: Front Cell Infect Microbiol. 2021 Oct 13;11:720357. doi: 10.3389/fcimb.2021.720357 (PMC8549493; doi:10.3389/fcimb.2021.720357)

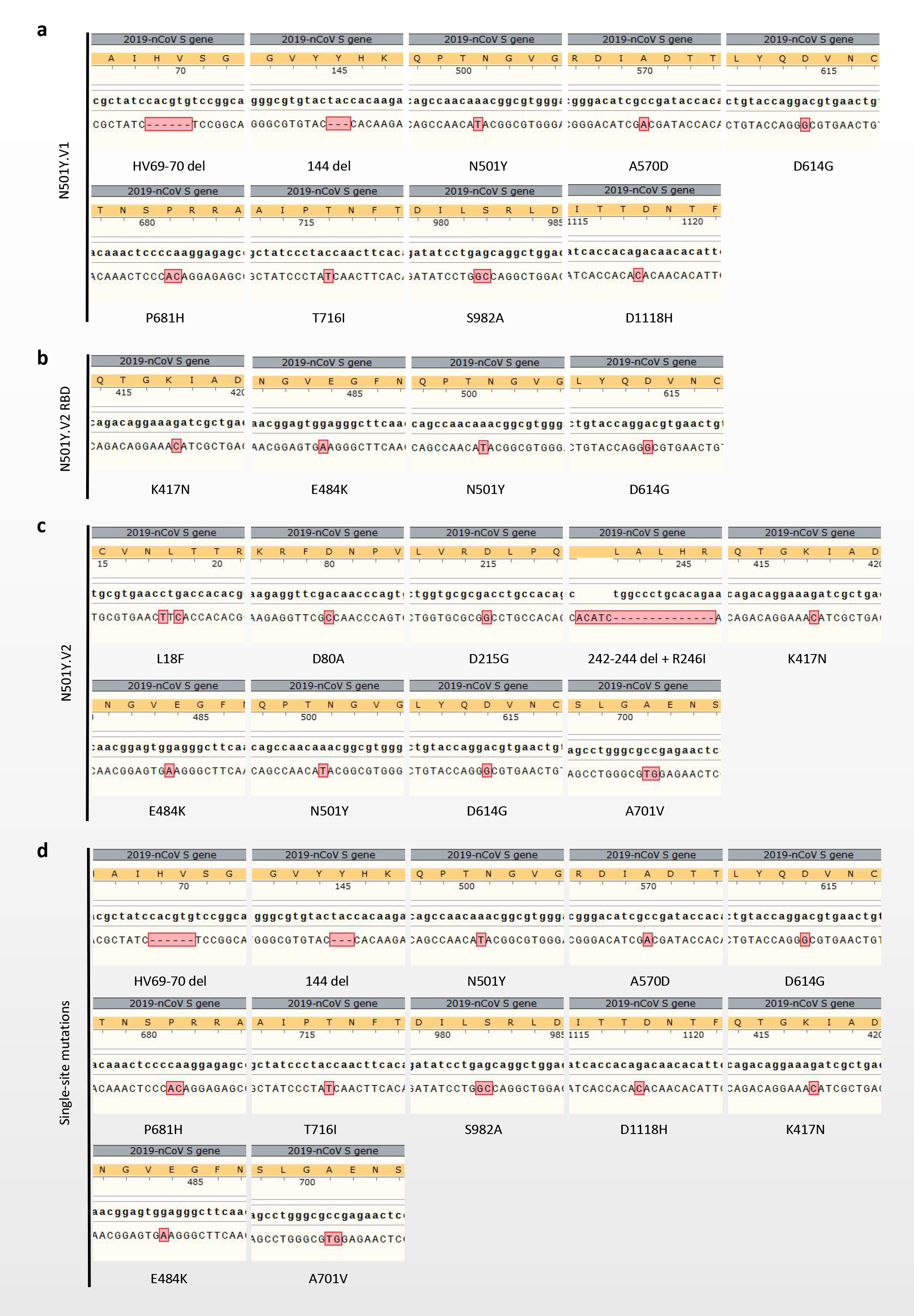

Supplement: Supplementary Figure 1 — DNA sequencing results of S gene mutations of N501Y.V1, N501Y.V2-RBD and N501Y.V2. SARS-CoV-2 S genes were sequenced with Sanger’s DNA sequencing at GenewiZ Biotechnology Co., Ltd. The DNA sequences of SARS-CoV-2 S mutants were compared with SARS-CoV-2 WT S gene with SnapGene software, and the related nucleotide mutation sites were marked in red boxes. [file Image_1.tif]

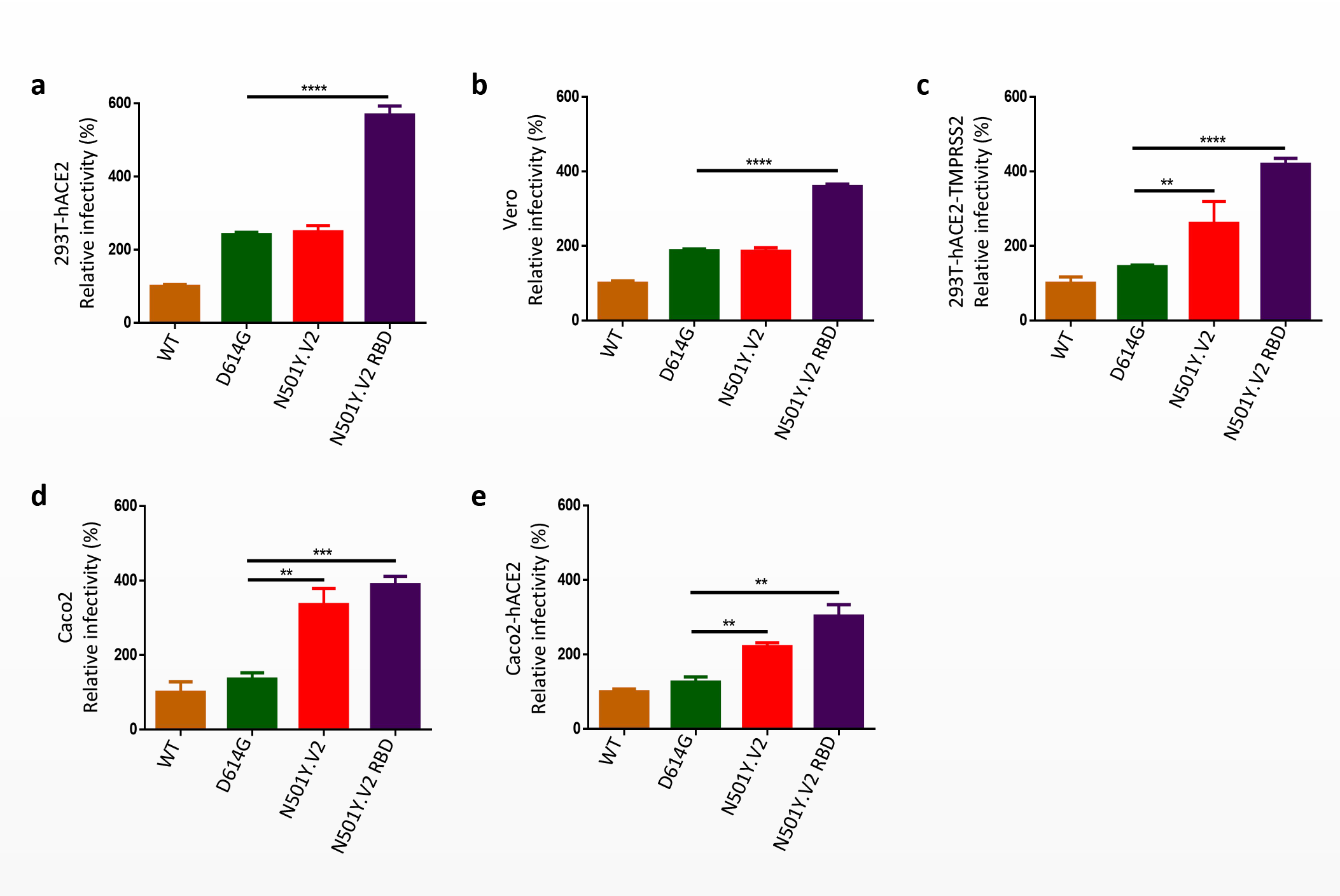

Supplement: Supplementary Figure 2 — Infection efficiency of SARS-CoV-2 N501Y.V2 in mammalian cell lines. Infection efficiency of SARS-CoV-2 WT, D614G, N501Y.V2 and N501Y.V2 RBD variants in 293T-hACE2 (A), Vero (B), 293T-hACE2-TMPRSS2 (C), Caco2 (D), Caco2-hACE2 (E) cells. ns represents no significant difference, p < 0.05 (*), p < 0.01 (**), p < 0.001 (***), p < 0.0001 (****). [file Image_2.tif]

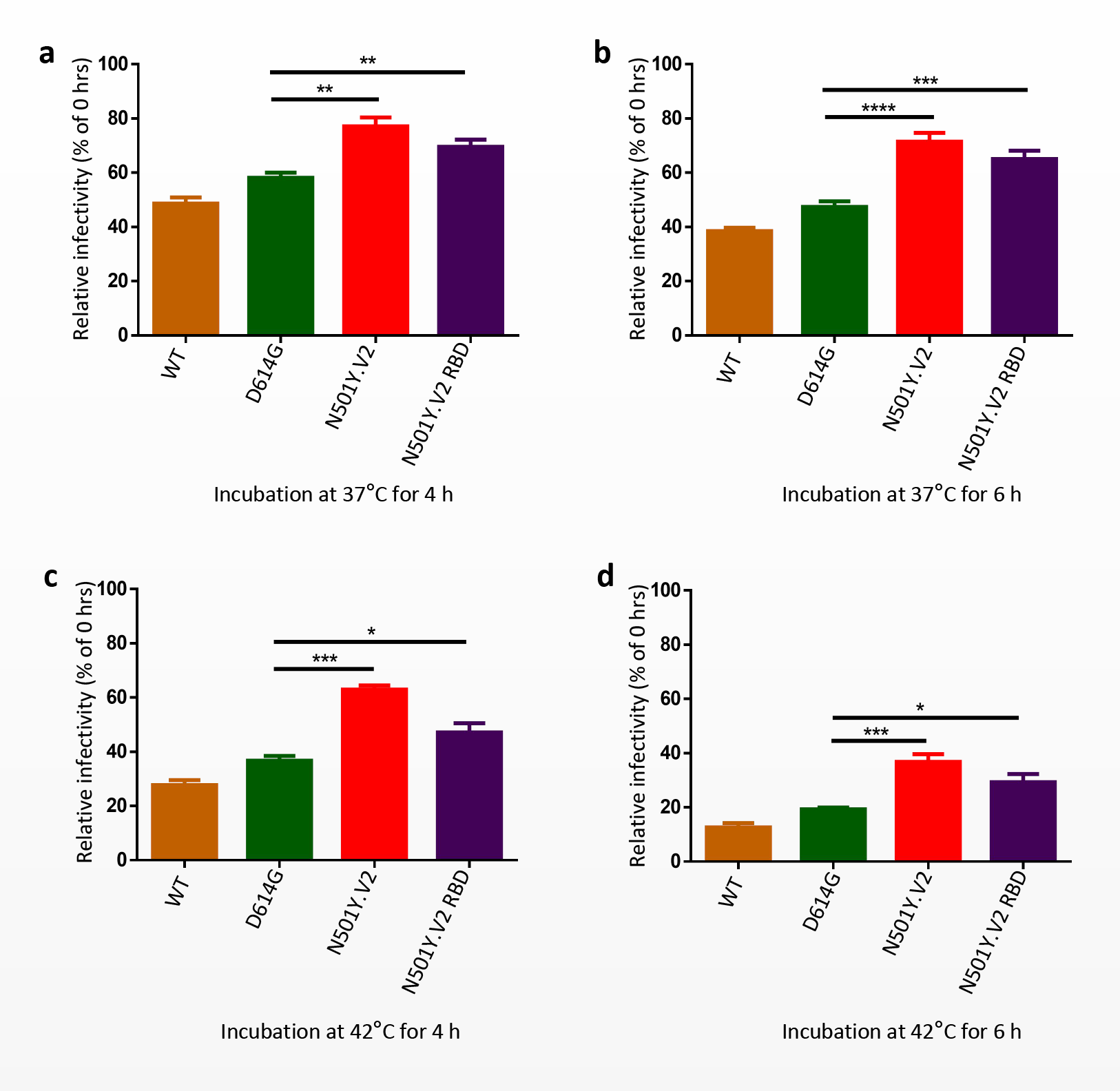

Supplement: Supplementary Figure 3 — SARS-CoV-2 N501Y.V2 is more thermal stable than WT and D614G. SARS-CoV-2 WT, D614G, N501Y.V2 and N501Y.V2 RBD S pseudovirions were incubated in cell culture medium DMEM at 37°C for 4 h (A) and 6 h (B) or 42°C for 4 h (C) and 6 h (D). The viruses were quantified for their infectious levels by luciferase on 293T-hACE2 cells. The infection efficiency of remaining viruses were normalized by the average fluorescence values at 0 h. ns represents no significant difference, p < 0.05 (*), p < 0.01 (**), p < 0.001 (***), p < 0.0001 (****). [file Image_3.tif]

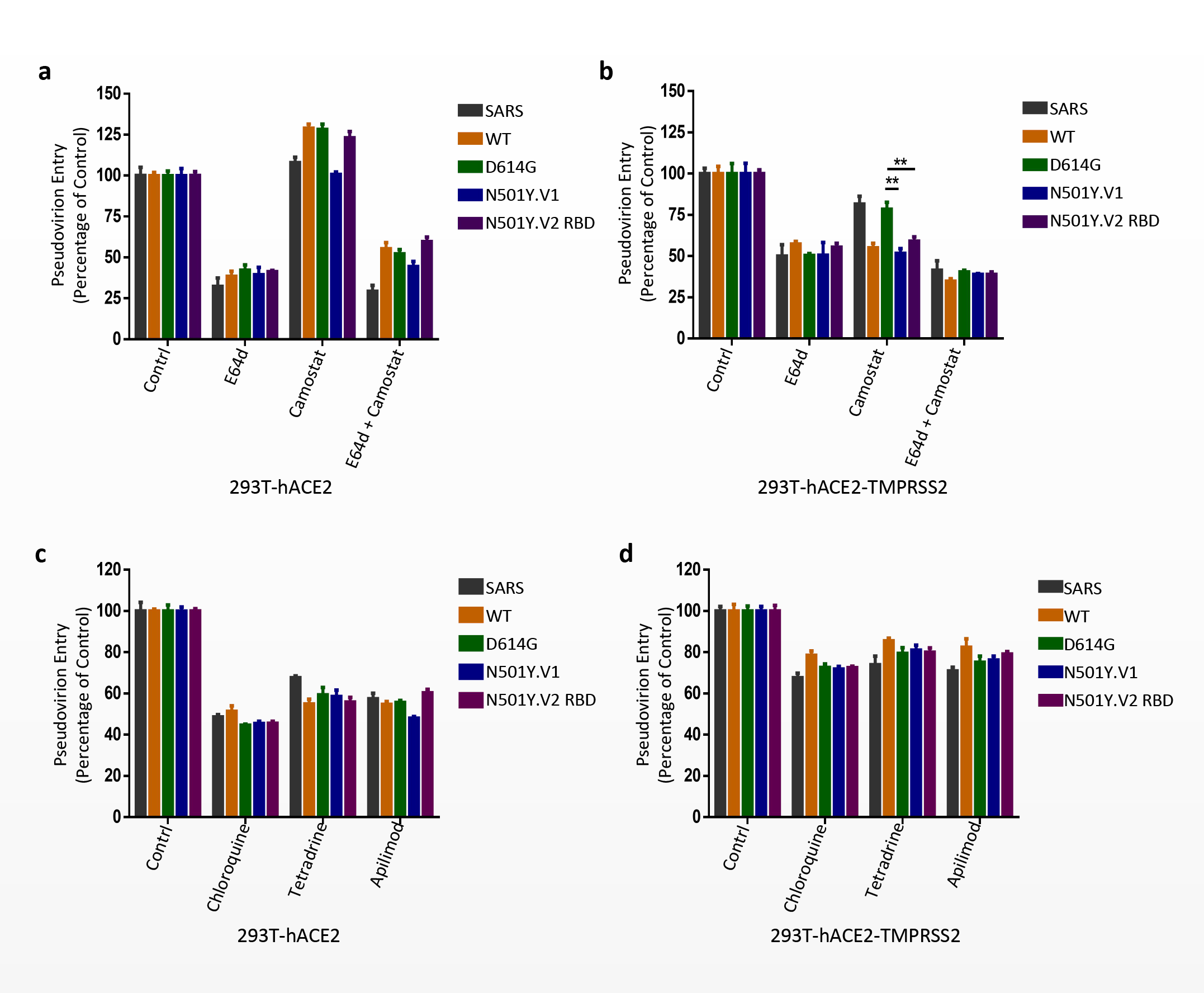

Supplement: Supplementary Figure 4 — The effects of protease and endocytosis pathway inhibitors on the entry of SARS-CoV-2 N501Y.V1 and N501Y.V2 RBD. (A, B) Effect of CatB/L or TMPRSS2 on SARS-CoV-2 N501Y.V1 and N501Y.V2-RBD entry into host cells. E64d (0.4 μM), Camostat (50 μM) or the combination of them (E64d (0.4 μM) + Camostat (50 μM)) were added into 293T-hACE2 (A) or 293T-hACE2-TMPRSS2 (B) cells 2 h prior to transduction. The luciferase activity was measured 24 h post transduction. (C, D) Effect of endocytosis on SARS-CoV-2 N501Y.V1 and N501Y.V2 RBD entry into host cells. Endocytosis inhibitors Chloroquine (1 μM), Tetradeine (0.2 μM), and Apilimod (5 nM) were added into 293T-hACE2 (C) or 293T-hACE2-TMPRSS2 (D) cells 2 h prior to transduction. Experiments were done in 4 replicates and repeated at least twice. One representative is shown with error bars indicating SEM. ns represents no significant difference, p < 0.05 (*), p < 0.01 (**), p < 0.001 (***), p < 0.0001 (****). [file Image_4.tif]
